# Intermediate Dose-Volume Parameters, Not Low-Dose Bath, Is Superior to Predict Radiation Pneumonitis for Lung Cancer Treated With Intensity-Modulated Radiotherapy

**DOI:** 10.3389/fonc.2020.584756

**Published:** 2020-10-15

**Authors:** Yinnan Meng, Wei Luo, Wei Wang, Chao Zhou, Suna Zhou, Xingni Tang, Liqiao Hou, Feng-Ming Spring Kong, Haihua Yang

**Affiliations:** ^1^ Laboratory of Cellular and Molecular Radiation Oncology, Radiation Oncology Institute of Enze Medical Health Academy, Affiliated Taizhou Hospital of Wenzhou Medical University, Taizhou, China; ^2^ Department of Radiation Oncology, Affiliated Taizhou Hospital of Wenzhou Medical University, Taizhou, China; ^3^ Department of Radiation Medicine, University of Kentucky, Lexington, KY, United States; ^4^ Department of Clinical Oncology, Hong Kong University Shenzhen Hospital and Queen Mary Hospital, Hong Kong University Li Ka Shing Medical School, Hong Kong, China; ^5^ Department of Radiation Oncology, University Hospitals/Seidman Cancer Center and Case Comprehensive Cancer Center, Case Western Reserve University, Cleveland, OH, United States

**Keywords:** lung cancer, intensity modulated radiotherapy (IMRT), radiation pneumonitis (RP), dosimetric parameters, prediction model

## Abstract

**Purpose:**

Although intensity-modulated radiotherapy (IMRT) is now a preferred option for conventionally fractionated RT in lung cancer, the commonly used cutoff values of the dosimetric constraints are still mainly derived from the data using three-dimensional conformal radiotherapy (3D-CRT). We aimed to compare the prediction performance among different dosimetric parameters for acute radiation pneumonitis (RP) in patients with lung cancer received IMRT.

**Methods:**

A total of 236 patients treated with IMRT were retrospectively reviewed in two independent groups of lung cancer from January 2014 to August 2018. The primary endpoint was grade 2 or higher acute RP (RP2). Dose metrics were generated from the bilateral lung volume outside GTV (Vdose_G_) and PTV (Vdose_P_). The associations of RP2 with clinical variables, dose-volume parameters and mean lung dose (MLD) were analyzed by univariate and multivariate logistic regression. The power of discrimination among each predictor was assessed by employing the bootstrapped area under the receiver operating characteristic curve (AUC), net reclassification improvement (NRI), and the integrated discrimination improvement (IDI).

**Results:**

Thirty-four (14.4%) out of 236 patients developed acute RP2 after the end of IMRT. The clinical parameters were identified as less important predictors for RP2 based on univariate and multivariate analysis. In both studied groups, the significance of association was more convincing in V20_P_, V30_P_, and MLD_P_ (smaller Ps) than V5_G_ and V5_P_. The largest bootstrapped AUC was identified for the V30_P_. We found a trend of better discriminating performance for the V20_P_ and V30_P_, and MLD_P_ than the V5_G_ and V5_P_ according to the higher values in AUC, IDI, and NRI analysis. To limit RP2 incidence less than 20%, the V30_P_ cutoff was 14.5%.

**Conclusions:**

This study identified the intermediate dose-volume parameters V20_P_ and V30_P_ with better prediction performance for acute RP2 than low-dose metrics V5_G_ and V5_P_. Among all studied predictors, the V30_P_ had the best discriminating power, and should be considered as a supplement to the traditional dose constraints in lung cancer treated with IMRT.

## Introduction

Acute radiation pneumonitis (RP), a challenging dose-limiting toxicity, commonly occurs within 1 to 6 months (most often within 12 weeks) after the completion of thoracic radiotherapy (RT) (1, 2). It is also the main reason to preclude the initiation of consolidative immunotherapy for local-advanced unresectable non-small cell lung cancer (3).

The quantitative analyses of normal tissue effects in the clinic (QUANTEC) lung project reviewed over 70 articles published before 2010 and provided the most reliable dose-RP relationship models to overcome the inconsistency (4). Accordingly the guidelines recommend the cutoff values of lung dose constraints to be the bilateral lung volume exceeding 20 Gy (V20) ≤35% and mean lung dose (MLD) ≤20 Gy (5, 6). However, the majority of evidence in the QUANTEC was based on three-dimensional conformal radiation therapy (3D-CRT), which may not well represent the dose distributions delivered by the more advanced techniques, such as intensity-modulated radiotherapy (IMRT). Ten years have passed since the QUANTEC, and there is a need to investigate more accurate dose predictors based on new data emerging from IMRT.

In a secondary analysis of the Radiation Therapy Oncology Group (RTOG) 0617, the IMRT group had a significantly larger V5 (61.6% vs. 58.4%), similar V20 and MLD compared to the 3D-CRT group. However, on the contrary, the severe RP was found to be significantly lower in the IMRT group (3.5% vs. 7.9%) (7). The commonly used dose constraints, especially V5, could not provide a sufficient explanation of why the severe RP was much lower in the IMRT group.

We hypothesized that the dose distribution differences between 3D-CRT and IMRT might impact the plan optimization strategy. This study aimed to analyze the prediction performance for symptomatic RP using various dosimetric parameters in two independent groups of lung cancer treated with IMRT.

## Materials and Methods

### Study Population

In this study, we retrospectively reviewed a total of 236 patients treated with IMRT between January 2014 and August 2018. The primary IMRT group included 183 consecutive patients with lung cancer treated before September 2017. The key inclusion criteria were pathologically confirmed lung cancer, available dosimetric data, follow-up records of at least three months, conventional daily fraction, first time receiving thoracic RT, and only thoracic IMRT. Patients receiving a prescription dose of less than 50 Gy were excluded from this study.

Starting from November 2017, we prescribed a higher dose for definitive radiotherapy to patients with unresectable stage III NSCLC. In addition, we routinely acquired a mid-treatment computed tomography (CT) and planned a new adaptive radiotherapy (ART). An independent group of 53 consecutive patients treated with IMRT-ART were selected using the same inclusion criteria as for the primary IMRT group.

The bilateral lung volumes were delineated according to the RTOG 1106 atlas of organs at risk under the revision and supervision of a senior physician (8). An additional lung definition was created for each patient by excluding the PTV from the bilateral lung volume. For all included patients, the collected clinical variables included age, gender, smoking status, tumor histology and stage, chemotherapy, and surgery. Dose metrics generated from dose-volume histograms (DVHs) in this study were including V5_G_, V10_G_, V20_G_, V30_G_, V40_G_, V50_G_, and MLD_G_ from the bilateral lung volume minus GTV, and V5_P_, V10_P_, V20_P_, V30_P_, V40_P_, V50_P_, and MLD_P_ from the lung minus PTV. The absolute lung volumes spared from certain dose levels were collected, including 5, 10, 20, 30, 40, and 50 Gy (AV5-50_Spared_). The total dose metrics for adaptative plans were summed up by using rigid registration and slightly manual adjustment with initial plans.

The institutional review board in our medical center waived the requirement of written informed consent because of the retrospective design in this study.

### Radiotherapy

Conventional or four-dimensional (4D) computed tomography (CT) was performed for the radiotherapy simulations. The patients were immobilized in the supine position with their arms above their head. The CT scans were performed with 5 mm or less slice thickness and included the entire neck and lung. Pre-treatment positron emission tomography (PET)/CT was not routinely used in staging and tumor volume delineation.

All of the patients were treated with conventionally fractionated simultaneous integrated boost IMRT. The gross tumor volume (GTV) was defined as the visible primary tumor and positive mediastinal lymph node on the treatment planning CT or pre-treatment PET scan. The clinical target volume (CTV) was defined as GTV with a 0.5 cm to 1 cm margin and the region at high risk for lymph node involvement. Another 5 mm uniform expansion was delineated from the GTV and CTV to create the planning gross tumor volume (PGTV) and planning target volume (PTV). Image guidance was performed with an orthogonal megavoltage electronic portal imaging device (EPID) or a kilovoltage cone beam computed tomography (CBCT) for inter-fractional geometric assurance.

The prescriptions of conventionally fractionated IMRT were 54 to 66 Gy to the PGTV and 45 to 54 Gy to the PTV in 25 to 30 fractions for curative intent. The prescription dose was 50 Gy to the PTV for the patients receiving postoperative RT with negative margins or local palliative purposes. All treatment plans were designed with the goal of delivering the prescribed dose to at least 95% of the PGTV and PTV.

### Endpoints and Follow-Ups

The primary endpoint was grade 2 or above acute radiation pneumonitis (RP2) within three months after radiotherapy. We graded RP according to the system described by Kong et al., which combines the considerations of SWOG, RTOG criteria, and CTCAE to provide an accurate assessment. The toxicities were prospectively evaluated during RT, and at 1 and 3 months of follow-up after the completion of IMRT. The diagnosis of acute RP was required to be distinguished from other causes, such as fibrosis, infection, or tumor recurrence.

### Statistical Analysis

For a description of the population, we used the median and range for continuous variables and the percentage for categorical variables. Univariate and multivariate logistic regression were performed to analyze the correlation between predictors and RP2. The age and location factors will be included in the multivariate analysis since they were found associated with a higher risk of pneumonitis from several previous reports (9, 10). All factors with a P value less than 0.20 in the univariate analysis will be included in a multivariate analysis. Because the multicollinearity among dose metrics, only one parameter at a time will be put in each multivariate model with set clinical factors. The patients who died before a diagnosis of RP2 were not censored, since only the acute phase of RP after radiotherapy was considered. The Akaike information criterion (AIC) and Bayesian information criterion (BIC) were applied to assess the relative goodness of fit for each dose prediction model. We employed the area under the receiver operating characteristic curve (AUC) of the receiver operating characteristic curve (ROC) to assess the RP2 discrimination performance, with the 1000-sample bootstrap method to internal validate the stability of the predictors. The RP2 risk predictors were further compared by the integrated discrimination improvement (IDI) and net reclassification improvement (NRI) analysis. A positive value of NRI or IDI indicates a preferred model over the reference in discriminating of the events and non-events (11, 12). Differences were considered significant at P<0.05 (2-sided). GraphPad Prism, version 8.02 (GraphPad Software, San Diego, California) and R (R Foundation for Statistical Computing, Vienna, Austria) were used in this study.

## Results

### Baseline Characteristics and RP2 Association Analysis

A total of 236 patients were retrospectively reviewed in this study. The clinical characteristics and their association with RP2 are shown in **Table 1**. RP2 was found in 34 patients (14.4%); 26 out of 183 (14.2%) in the IMRT group, and 8 out of 53 (15.1%) in the IMRT-ART group. In the univariate logistic regression, none of the clinical factors was significantly associated with RP2, although the female gender (P=0.101) and the use of chemotherapy (P=0.107) had a trend of higher RP2 risk.

**Table 1:** Correlation of clinical characteristics with grade ≥2 acute radiation pneumonitis.

Characteristics	No. of Patients(N=236) (%)	No. of Grade ≥2 RP(N=34) (%)	Odds Ratio	95% CI	P Value*
Age					
≤64 (Median)	125 (53)	17 (50.0)	1.00		
>64 (Median)	111 (47)	17 (50.0)	1.15	0.56–2.38	0.708
Gender					
Male	213 (90.3)	28 (82.0)	1.00		
Female	23 (9.7)	6 (18.0)	2.33	0.85–6.42	0.101
Smoking					
Non-smoker	47 (19.9)	7 (21.0)	1.00		
Smoker	189 (80.1)	27 (79.0)	0.95	0.39–2.34	0.915
Pathology					
Squamous	158 (66.9)	25 (74.0)	1.00		
Adenocarcinoma	36 (15.3)	5 (15.0)	0.86	0.30–2.42	0.772
Small Cell	36 (15.3)	3 (9.0)	0.48	0.14–1.70	0.257
Others	6 (2.5)	1 (3.0)	1.06	0.12–9.50	0.956
Stage					
I/II	14 (5.9)	0 (0)	0		0.999
III	168 (71.2)	28 (82)	1.60	0.63–4.10	0.327
IV	54 (22.9)	6 (18)	1.00		
Tumor location					
Upper	109 (46.2)	14 (41.2)	1.00		
Middle or lower	127 (53.8)	20 (58.8)	1.27	0.61–2.65	0.527
Chemo					
No	29 (12.3)	1 (3.0)	1.00		
Yes	207 (87.7)	33 (97.0)	5.31	0.70–40.40	0.107
Surgery					
No	181 (76.7)	28 (82.4)	1.00		
Yes	55 (23.3)	6 (17.6)	0.67	0.26–1.71	0.401

RP, radiation pneumonitis; CI, confidence interval.

*By univariate logistic regression analysis.

**Figure 1:**
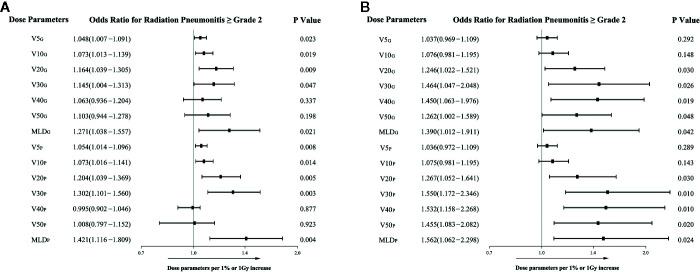
The associations of dosimetric parameters with grade ≥2 radiation pneumonitis in the univariate logistic regression analysis. **(A)** The associations in the primary IMRT group; **(B)** The associations in the IMRT-ART group.

In the primary IMRT group, a significant association with RP2 was found for V5, V10, V20, V30, and MLD from two lung definitions. The significance was more convincing in V20_P_ (P= 0.005, OR=1.204), V30_P_ (P=0.003, OR=1.302) and MLD_P_ (0.004, OR=1.421) than in the other parameters (**Figure 1A**). In the IMRT-ART group, only the parameters of V20, V30, and MLD from both lung volumes were confirmed to be significantly associated with RP2 (**Figure 1B**).

In the multivariate analysis, age, tumor location, and chemotherapy did not reach significance (All Ps> 0.05). All of the dosimetric factors remained as independent predictors of RP2 in each of their multivariate models. Female gender was found significantly associated with RP2 in the models including V20_G_ and V20_P_, but not had a significant association in those including other dosimetric parameters (**Table S1**). Given a very limited number of female patients were included in this study (9.7%), the gender factor will not be considered in the direct comparison of the prediction performance for RP2 using different dosimetric factors. Discrimination performance for RP2

We employed the bootstrapped area under the ROC (AUC) to evaluate the discrimination performance for RP2 using each dosimetric parameter in 236 patients. The V30_P_ had the best prediction performance among all dose metrics (AUC=0.683). We found that the V5, V20, V30 and MLD from the bilateral lung volume minus PTV with larger AUCs than the ones from the lung minus GTV. The V20, V30 and MLD from both lung volumes showed a trend of better discriminating values than V5, even their confidence intervals of AUCs overlapped. The absolute volume of spared lung parameters showed lower prediction values for RP2 (All AUCs smaller than 0.55) compared with the dose-volume predictors (**Table 2**).

**Table 2:** Bootstrapped AUC and 95% CI for dosimetric parameters.

Parameters	AUC	Lower CI	Upper CI
V5_G_	0.603	0.538	0.666
V10_G_	0.634	0.550	0.678
V20_G_	0.650	0.585	0.711
V30_G_	0.623	0.558	0.685
V40_G_	0.596	0.530	0.659
V50_G_	0.579	0.513	0.643
MLD_G_	0.638	0.573	0.699
V5_P_	0.615	0.550	0.678
V10_P_	0.643	0.578	0.704
V20_P_	0.650	0.585	0.710
V30_P_	0.683	0.620	0.742
V40_P_	0.619	0.553	0.681
V50_P_	0.579	0.513	0.643
MLD_P_	0.677	0.613	0.736
AV5_Spared_	0.513	0.447	0.578
AV10_Spared_	0.506	0.440	0.571
AV20_Spared_	0.522	0.456	0.587
AV30_Spared_	0.535	0.469	0.600
AV40_Spared_	0.539	0.474	0.604
AV50_Spared_	0.550	0.484	0.614

AUC, the area under the receiver operating curves; RT, radiotherapy; Vdose_G_, MLD_G_, dosimetric parameters from lung volume excluding gross tumor volume; Vdose_P_, MLD_P_, dosimetric parameters from lung volume excluding planning treatment volume; AVdose_spared_, Absolute volume of lung spared above certain threshold of dose; V5–50, volume of lung receiving a dose≥5–50Gy; MLD, mean lung dose; CI, confidential interval.

**Figure 2:**
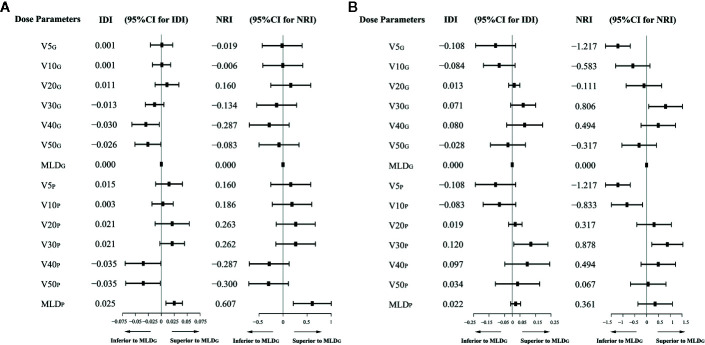
Integrated discrimination improvement (IDI) and net reclassification improvement (NRI) analysis for each dosimetric predictor compared with the MLD_G_. **(A)** IDI and NRI values in the primary IMRT group; **(B)** IDI and NRI values in the IMRT-ART group.

The V20_P_, V30_P_, and MLD_P_ displayed a trend towards larger values of NRI and IDI than the most commonly used parameter, MLD_G_, in both the primary IMRT and IMRT-ART groups. The V5 and V10 from two lung volumes compared with the MLD_G_ had less reliable prediction performance in IMRT-ART group, while V40 and V50 were significantly inferior in discrimination based on the primary IMRT data. Details of NRI and IDI analysis for each dose metrics are shown in **Figure 2**.

### Evaluation of the Goodness of Fit for Prediction Models

**Figure 3:**
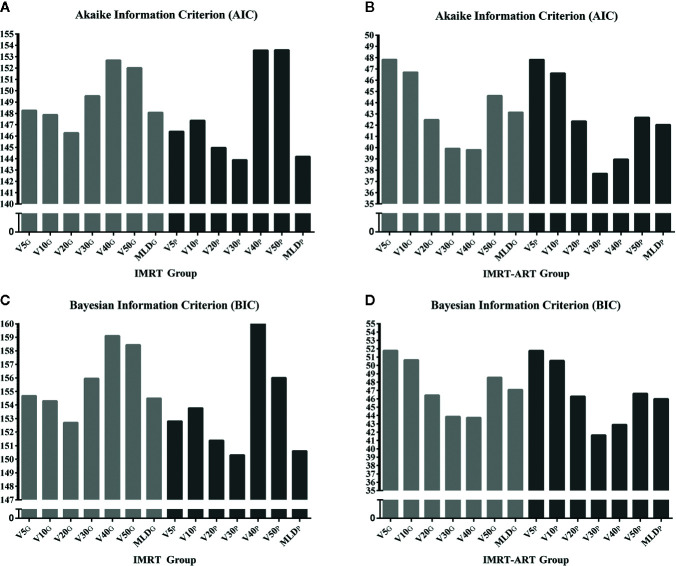
The relative evaluation of goodness of fit test for a model selection using Akaike information criterion (AIC) and Bayesian information criterion (BIC). **(A)** The relative values of the AIC in the IMRT group; **(B)** The relative values of the AIC in the IMRT-ART group; **(C)** The relative values of the BIC in the IMRT group; **(D)** The relative values of the BIC in the IMRT-ART group.

The Akaike information criterion (AIC) and Bayesian information criterion (BIC) were used to evaluate the relative values of the goodness of fit for RP2 prediction models in two independent groups. Among all candidate models, better data fitness with the smallest values of AIC and BIC were found in the model with V30_P_ in both IMRT and IMRT-ART groups. The models with V20_P_, V30_P,_ and MLD_P_ had relatively smaller values of AIC and BIC than V5_G_ and V5_P_ in both groups of patients (**Figure 3**).

#### Prediction Model With V30_P_

**Figure 4:**
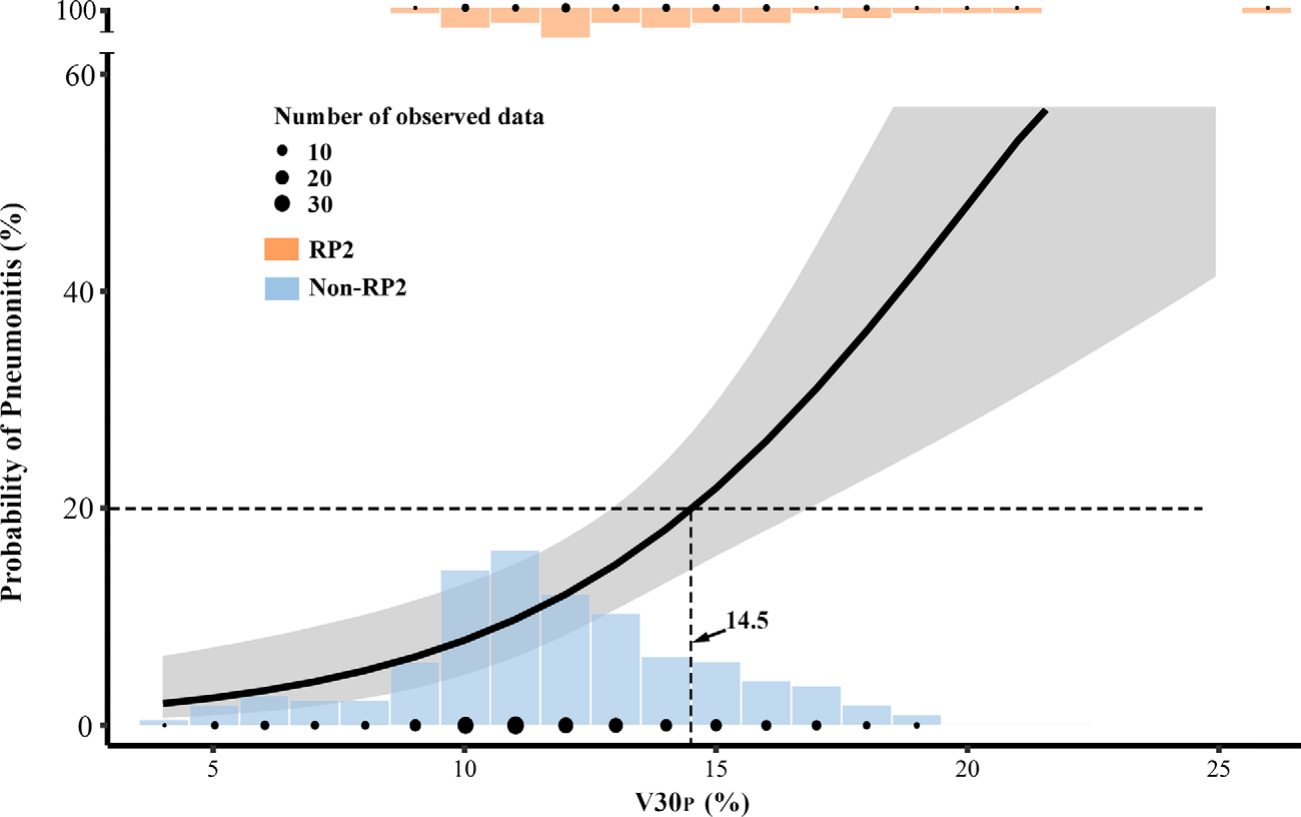
The prediction model with V30_P_ was plotted in a solid curve with a 95% confidential interval for the probability of grade≥2 acute radiation pneumonitis (RP2). The V30_P_ cutoff was 14.5% for limiting 20% RP2. The plotted dots and columns represented the number of observed data at each dose level.

The probability of RP2 from 236 included patients can be estimated from V30_P_ by a fitted logistic formula: Logit(P) = −4.84+0.238X; P (% of RP2) =1/[1+exp (−0.238*V30_P_ + 4.84)]. The prediction curve was plotted in **Figure 4**. The Hosmer-Lemeshow test showed no significant departure from a well-fitted model (P=0.968). To limit the probability of RP2 less than 20%, the V30_P_ should be controlled to under 14.5%. According to current data, when the V30_P <_14.5%, the RP2 incidence was 11.2%; and when the V30_P >_14.5%, the RP2 incidence was 26.5%.

## Discussion

This study demonstrated that the V30_P_ had the best RP2 prediction performance among all dosimetric parameters in two independent groups. None of the clinical factors showed a significant correlation with RP2 in univariate and multivariate analysis. The best prediction performance for RP2 was found with V30_P_, based on better goodness of fit in AIC and BIC, the largest bootstrapped AUC, and an upward trend towards higher NRI and IDI compared to other dosimetric predictors.

To the best of our knowledge, when using IMRT, this is one of the first studies to compare the prediction performance for RP2 among dosimetric factors from two lung definitions. The results showed that putting a higher priority on the V30_P_ over V5 in planning optimization may better shape the DVHs, which may lead to a lower RP2 probability.

The normal lung volume definition currently recommended by both the RTOG and EORTC guidelines is total bilateral lung volume minus GTV (5, 8). QUANTEC also recommended using the definition of excluding GTV for the consideration of inconsistent delineation of the PTV from one institution to another (4). Therefore, most studies using IMRT from the past several years only report dose data from the lung volume minus GTV (13–15). However, our previous studies demonstrated that the dosimetric parameters from the lung minus PTV are not inferior to those in the lung minus GTV for RP2 prediction (16). In this study, we further demonstrated that V30_P_ might be the best predictor among all parameters. Reducing the lung dose inside the PTV may not be reasonable during the optimization process. The conflict between getting 95% of the PTV covered with prescription and simultaneously reducing the dose to the lung in the PTV may complicate the optimization process.

Various dosimetric parameters are highly correlated with each other (17–19). The relative priority of reducing one dose parameter at the expense of increasing another is still unknown. However, with IMRT and VMAT, we have more freedom to optimize the shape of the DVH by reducing the volume of the intermediate dose region by irradiating a more substantial volume with a low dose bath. A few studies discussed the question of whether to deliver a low dose to a larger volume (“a little to a lot”) or a high dose to a smaller volume (“a lot to a little”) to further reduce the symptomatic RP probability. Willner et al. concluded that a small dose to a large volume was preferable to a large dose to a small lung volume (20). Multiple studies, on the other hand, highlighted the importance of the V5 or other low dose predictors. Metha et al. argued that “a little to a lot” could be worse than “a lot to a little” because the loss of carbon monoxide diffusing capacity occurs at 13 Gy (21). Wang et al. analyzed 223 patients treated with 3D-CRT and found a cutoff point of 42% in the V5 to have the best discrimination power for severe RP (18). Yorke et al. concluded that the low dose from V5 to V13 in the total and ipsilateral lung volume were more strongly correlated with severe RP than the V20 and higher dose parameters (19). In the IMRT era, however, the lung V5 did not show a higher priority than other dose metrics for lung toxicity prevention based on most studies (7, 22). Tucker et al. analyzed the differences in RP risk for patients receiving the same MLD but with different shapes of the DVHs. They suggested that the high dose region plays a more important role than the mean lung dose in the risk of severe RP; “a lot to a little” is associated with a higher risk of severe RP than “a little to a lot.” (23) These findings were also confirmed in their later validation study (24). Our results found an inferior predictive value for the V5, which was consistent with the IMRT studies above. We further demonstrated a better RP2 prediction performance with the V30 in the lung region outside the PTV. Adding V30_P_ along with the traditional V20_G_ and MLD_G_ constraints in treatment planning optimization may better shape the DVH to further reduce the RP2 probability.

We recognized that this study is limited in several aspects. First, the patients in the IMRT- ART group had a mid-treatment CT scan and a treatment replanning. The difference in dose calculation methods between non-adaptive and adaptive RT could have resulted in variation in the overall lung dose estimation. However, the prediction performance for each dose parameter was always directly compared in a single patient. Different approaches could have impacted the exact cutoff value, but they would not have changed the relative predictive power regarding which predictor is better. Second, this was a single-institution retrospective study; 236 patients were still a small sample size considering that only 34 patients developed acute RP2. Third, immunotherapy after definitive concurrent chemoradiotherapy is considered a standard routine practice for unresectable locally advanced NSCLC (25). However, during the time of this study, our patients did not have access to PD-1 or PD-L1 inhibitors. The influence of immunotherapy on RP toxicity was not considered in this study.

Ideally, the individual sensitivity to RP2 should be identified before determining the RT prescription. Some investigators have focused on the impact of clinical factors on RP (9, 26, 27). In the current study, only the gender and chemotherapy showed a trend of association with RP2. The older age and lower lobe location were not identified as high-risk factors, and they may not significantly impact the comparison results of dosimetric predictors. Correlations between biological markers and an increased risk of RP have also been found in several studies (28–30). However, none of these risk predictors has been applied and proved in a prospective clinical trial yet. The conventionally fractionated definitive RT for lung cancer is generally prescribed at 60 to 70 Gy with no further escalation in routine practice (31, 32). The most important tool to prevent symptomatic RP is not only to keep it under “safety” criteria, but also to optimize the lung dose as low as reasonably. Our results suggested that the V30_P_ should be weighted as a higher priority dose constraint in the treatment planning optimization in order to lower the RP2 risk further. A large external dataset from other institutions is needed in the future to further validate the superior RP2 predictive value of the V30 from the lung volume outside the PTV.

## Data Availability Statement

The raw data supporting the conclusions of this article will be made available by the authors, without undue reservation.

## Ethics Statement

The studies involving human participants were reviewed and approved by the institutional review board of Taizhou Hospital. Written informed consent for participation was not required for this study in accordance with the national legislation and the institutional requirements.

## Author Contributions

HY, F-MSK, and WL had the ideas and contributed to the study design. YM, WW, HY, and LH carried out the statistical analysis. YM, CZ, SZ, WL, and WW contributed to the literature search. YM, LH, and XT contributed to the treatment planning and dosimetry data. CZ, SZ, and HY contributed to the evaluation of radiation pneumonitis and clinical data. YM, WL, F-MSK, and HY were major contributors to the writing of the manuscript. All authors contributed to the article and approved the submitted version.

## Funding

This work was funded by the National Natural Science Foundation of China (81874221), and Taizhou Science and Technology Bureau (20ywa09 and 1802ky07).

## Conflict of Interest

The authors declare that the research was conducted in the absence of any commercial or financial relationships that could be construed as a potential conflict of interest.
