# Supplementary material for: Intermediate Dose-Volume Parameters, Not Low-Dose Bath, Is Superior to Predict Radiation Pneumonitis for Lung Cancer Treated With Intensity-Modulated Radiotherapy
Source: Front Oncol. 2020 Oct 15;10:584756. doi: 10.3389/fonc.2020.584756 (PMC7594624; doi:10.3389/fonc.2020.584756)
Supplement: Supplementary file 1 [file DataSheet_1.docx]

| Table S1. Multivariate models including each dosimetric parameter with selected clinical variables | | | |
| --- | --- | --- | --- |
| Variables | Odds Ratio | 95% CI | P Value |
| Model including V5_G_ | 1.043 | 1.004-1.084 | 0.031 |
| Age | 1.576 | 0.722-3.440 | 0.254 |
| Gender | 2.458 | 0.837-7.218 | 0.110 |
| Chemo | 5.364 | 0.685-41.987 | 0.102 |
| Location | 0.907 | 0.393-2.091 | 0.819 |
| Model including V5_P_ | 1.049 | 1.010-1.089 | 0.014 |
| Age | 1.612 | 0.735-3.534 | 0.233 |
| Gender | 2.38 | 0.801-7.070 | 0.118 |
| Chemo | 5.217 | 0.665-40.903 | 0.116 |
| Location | 0.838 | 0.359-1.956 | 0.683 |
| Model including V20_G_ | 1.178 | 1.070-1.297 | 0.001 |
| Age | 1.652 | 0.742-3.676 | 0.219 |
| Gender | 3.133 | 1.043-9.409 | 0.042 |
| Chemo | 5.058 | 0.630-40.633 | 0.127 |
| Location | 0.98 | 0.446-2.154 | 0.960 |
| Model including V20_P_ | 1.189 | 1.077-1.313 | 0.001 |
| Age | 1.581 | 0.714-3.501 | 0.259 |
| Gender | 3.212 | 1.067-9.663 | 0.038 |
| Chemo | 4.662 | 0.583-37.292 | 0.147 |
| Location | 1.033 | 0.472-2.262 | 0.935 |
| Model including V30_G_ | 1.179 | 1.054-1.319 | 0.004 |
| Age | 1.553 | 0.705-3.421 | 0.275 |
| Gender | 2.737 | 0.926-8.085 | 0.069 |
| Chemo | 4.87 | 0.616-38.518 | 0.134 |
| Location | 1.213 | 0.563-2.613 | 0.622 |
| Model including V30_P_ | 1.265 | 1.115-1.435 | < 0.001 |
| Age | 1.652 | 0.737-3.706 | 0.223 |
| Gender | 2.767 | 0.919-8.327 | 0.07 |
| Chemo | 4.172 | 0.528- 32.988 | 0.176 |
| Location | 1.261 | 0.577-2.752 | 0.561 |
| Model including MLD_G_ | 1.292 | 1.090-1.533 | 0.003 |
| Age | 1.595 | 0.722-3.523 | 0.248 |
| Gender | 2.775 | 0.934-8.239 | 0.066 |
| Chemo | 4.993 | 0.629-39.643 | 0.128 |
| Location | 1.001 | 0.456-2.196 | 0.999 |
| Model including MLD_P_ | 1.443 | 1.184-1.760 | < 0.001 |
| Age | 1.69 | 0.755-3.784 | 0.202 |
| Gender | 2.862 | 0.950-8.624 | 0.062 |
| Chemo | 4.691 | 0.588-37.449 | 0.145 |
| Location | 0.894 | 0.401-1.993 | 0.784 |

Abbreviation: Vdose_G_, dosimetric parameters from lung volume excluding gross tumor volume; Vdose_P_, dosimetric parameters from lung volume excluding planning treatment volume; V5-30, volume of lung receiving a dose≥5-30Gy; MLD, mean lung dose; CI, confidential interval.
